# Differing patterns in thermal injury incidence and hospitalisations among 0–4 year old children from England

**DOI:** 10.1016/j.burns.2016.05.007

**Published:** 2016-11

**Authors:** Ruth Baker, Laila J. Tata, Denise Kendrick, Tiffany Burch, Mary Kennedy, Elizabeth Orton

**Affiliations:** a Division of Primary Care, The University of Nottingham, Tower Building, University Park, Nottingham NG7 2RD, United Kingdom; b Division of Epidemiology and Public Health, The University of Nottingham, Clinical Sciences Building, Nottingham City Hospital, Hucknall Road, Nottingham NG5 1PB, United Kingdom; c Nottingham Burns Unit, Nottingham University Hospitals, NHS Trust, Nottingham City Hospital, Hucknall Road, Nottingham NG5 1PB, United Kingdom

**Keywords:** Burns, Children, Epidemiology, Thermal injuries, England

## Abstract

**Objective:**

To describe patterns in thermal injury incidence and hospitalisations by age, gender, calendar year and socioeconomic status among 0–4 year olds in England for the period 1998–2013.

**Participants:**

708,050 children with linked primary care and hospitalisation data from the Clinical Practice Research Datalink (CPRD) and Hospital Episode Statistics (HES), respectively.

**Analysis:**

Incidence rates of all thermal injuries (identified in CPRD and/or HES), hospitalised thermal injuries, and serious thermal injuries (hospitalised for ≥72 h). Adjusted incidence rate ratios (IRR) and 95% confidence intervals (95%CI), estimated using Poisson regression.

**Results:**

Incidence rates of all thermal injuries, hospitalised thermal injuries, and serious thermal injuries were 59.5 per 10,000 person-years (95%CI 58.4–60.6), 11.3 (10.8–11.8) and 2.15 (1.95–2.37), respectively. Socioeconomic gradients, between the most and least deprived quintiles, were steepest for serious thermal injuries (IRR 3.17, 95%CI 2.53–3.96). Incidence of all thermal injuries (IRR 0.64, 95%CI 0.58–0.70) and serious thermal injuries (IRR 0.44, 95%CI 0.33–0.59) reduced between 1998/9 and 2012/13. Incidence rates of hospitalised thermal injuries did not significantly change over time.

**Conclusion:**

Incidence of all thermal injuries and those hospitalised for ≥72 h reduced over time. Steep socioeconomic gradients support continued targeting of preventative interventions to those living in the most deprived areas.

## Highlights

• Thermal injury incidence based on linked data reduced from 1998 to 2013 in 0–4 year olds.
• Incidence of hospitalised thermal injuries did not significantly change over time.
• Inequalities between the most and least deprived areas narrowed over time.
• Socioeconomic gradients were steepest for serious thermal injuries.

## 1 Background

Thermal injuries (e.g. hot water scalds, flame burns) cause morbidity, prolonged hospitalisation and disability in children aged 0–4 years old both globally and within the United Kingdom (UK). They are the fourth leading cause of injury-related hospitalisation among 0–4 year olds in England [1], and were highlighted in 2014 by Public Health England as one of the five priority injuries for prevention in this age group [1]. Serious burns and scalds have a significant impact on the child, family and health services and can lead to high treatment costs (e.g. £173,000 to treat a serious bathwater scald [2]). Among young children thermal injuries most commonly occur within the home and are largely preventable.

Quantifying the burden of thermal injuries in England is a challenge, with existing national data focusing on those undergoing hospitalisation [3] or specialist burns care [4]; representing a small proportion of the overall burden of thermal injury. Within England over 98% of the population is registered with a general practitioner (GP) [5], with GPs maintaining longitudinal electronic records of patients’ medical conditions, including recording diagnoses made in secondary and tertiary care. Through using the Clinical Practice Research Datalink (CPRD), a nationally representative primary care database that is linked to hospitalisation data, we aimed to describe patterns in thermal injury incidence and hospitalisations by age, gender, calendar time and socioeconomic status amongst a cohort of children aged 0–4 years from England.

## 2 Methods

### 2.1 Study population

The CPRD is a primary care research database containing the longitudinal primary care records of over 11 million patients from the UK [6]. It has been validated for a number of diseases [7] and is broadly representative of the demographics of the UK population [6]. We used the CPRD to yield a study population of 708,050 children from England, who were aged 0–4 years old between 1st January 1998 and 31st December 2013 and for whom linked hospitalisation data were available. Hospitalisation data are held in the Hospital Episode Statistics (HES) inpatient dataset, which captures all elective and emergency hospitalisations paid for by the National Health Service (NHS). Linked hospitalisation records are available for 75% of English CPRD practices [6], and have been shown to broadly represent the age and gender structure of the English population [6], [8], but underrepresent some regions (North East, East Midlands and Yorkshire and the Humber) [8].

Using the CPRD, we carried out an open cohort study, with children entering the cohort at the latest date of: their date of birth, their general practice registration date, 1st January 1998, and the date the practice met the CPRD data quality standards. Each child contributed data to the study until their end of follow-up date, which was the earliest of: 31st December 2013, the child's fifth birthday, the date medical data were last collected from the general practice, or the date the child left the practice (e.g. child moved practice or died). The study population was therefore a subset of children from England, representing approximately 6% of 0–4 year olds from England in 2013.

### 2.2 Identification of thermal injury records

For each child in the study cohort we identified any recorded thermal injury events occurring during their follow-up time from their primary care (CPRD) and/or hospitalisation records (HES). The CPRD contains information about thermal injuries managed in primary care, but also contains information communicated to the GP about emergency department (ED) attendances and hospitalisations. Previous studies have shown high levels of transcription of information from discharge letters and outpatient summaries into the primary care record [9], [10]. Diagnoses are recorded in the CPRD using Read Codes, a clinical coding system used in UK primary care. We identified thermal injuries recorded in the CPRD using a list of Read codes (Supplementary file 1), corresponding to International Classification of Diseases version 10 (ICD-10) categories for burns (ICD-10 T20-T32), injuries due to heat and hot substances (ICD-10 X10-X19), and injuries due to smoke, fire and flames (ICD-10 X00-X09). Chemical burns, corrosions and abrasion burns were excluded. We identified hospitalisations for thermal injuries by extracting any records from HES with an ICD-10 code (T20-T32, X10-X19, X00-X09) or procedure code (e.g. codes for dressing, debridement or exploration of burnt skin, skin grafts) for a thermal injury.

### 2.3 Identifying incident thermal injuries

To identify incident events using both CPRD and HES data, it was necessary to exclude duplicate records for the same injury recorded in both data sources, and to exclude repeat records for the same injury event (e.g. repeated dressing changes). We did this by using a time-based algorithm (Supplementary file 2), as previously described [11]. In brief, we assessed the time between the first code for a thermal injury event and all subsequent thermal injury codes. Primary care records that occurred within 3 weeks of the event date, if the event was first recorded in primary care, or 8 weeks of the event date if the first record was a hospitalisation, were considered the same event. A longer time-window was used for thermal injuries undergoing hospitalisation as these are likely to be more severe injuries and benefit from longer follow-up. A third time-window of six weeks determined whether hospitalisations occurring after the event start date referred to the same (e.g. readmission) or a new event. Thermal injury codes occurring outside of these time-windows were considered a new injury event. To account for a small number of children receiving repeated skin grafts, any codes for grafts occurring within two years of the first thermal injury event code were considered the same event. We identified these time-windows by plotting the rates of thermal injury codes entered in CPRD and HES after the first injury code [11], and have previously demonstrated that even when these time-windows are doubled, incidence rates by child age are similar to the primary analysis [11].

For each child we also identified the number of incident thermal injury events that underwent hospitalisation and the number of ‘serious’ thermal injury events. We defined serious thermal injuries as those undergoing hospitalisation for 72 h or more, a definition previously used in a study of traumatic injury [12], as within the CPRD and HES databases there is insufficient data coded to accurately assess injury severity.

### 2.4 Statistical analyses

We estimated incidence rates per 10,000 person-years (PY), with 95% confidence intervals (95%CI), for thermal injuries identified in CPRD and/or HES, those undergoing hospitalisation, and serious thermal injuries (hospitalised for 72 h or more), by child age, gender, socioeconomic status and calendar time. Socioeconomic status was measured using quintiles of the Index of Multiple Deprivation 2010, an area based measure of deprivation based upon 38 indicators covering income, employment, health, education, crime, access to services and the living environment. Adjusted incidence rate ratios (IRRs) were estimated using Poisson regression, mutually adjusting for child age, gender, socioeconomic status, region and calendar year. We tested for an interaction between socioeconomic status and calendar year using a likelihood ratio test, with *p* < 0.01 considered statistically significant.

To estimate the burden of thermal injuries among children aged 0–4 years old for the whole of England in 2013, we applied our 2013 incidence rates by age and gender to the 2013 mid-year population estimate for England [13]. In addition, to allow comparison of our data with that from a previous surveillance system, the Home and Leisure Accident Surveillance System (HASS/LASS), that operated in the UK until 2002 [14] we estimated the number of thermal injuries occurring between 1998 and 2002 in the UK by applying our estimated incidence rates to the mid-year population estimates for each of these years.

This study was approved by the Independent Scientific Advisory Committee for the Medicines and Healthcare products Regulatory Agency (protocol 13-199R), giving permission for use of both CPRD and HES data.

## 3 Results

The study population consisted of 708,050 children aged 0–4 years old who were contributing to the CPRD database between 1998 and 2013, registered across 393 general practices. Of these children, 369,513 (52.2%) were male and 338,537 (47.8%) were female (Table 1). Median length of follow-up per child was 2.5 years (interquartile range 1.1–4.7 years).

### 3.1 Incidence rates of thermal injuries

**Fig. 1:**
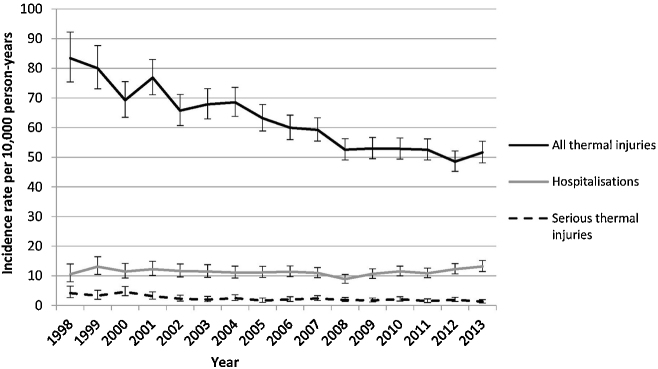
Thermal injury incidence, hospitalisations, and hospitalisations undergoing admission for 72 h or more, among children aged 0–4 by calendar year. ‘All thermal injuries’ include all events identified in primary care and/or hospitalisation data. Serious thermal injuries were defined as those undergoing hospital admission for ≥72 h.

**Fig. 2:**
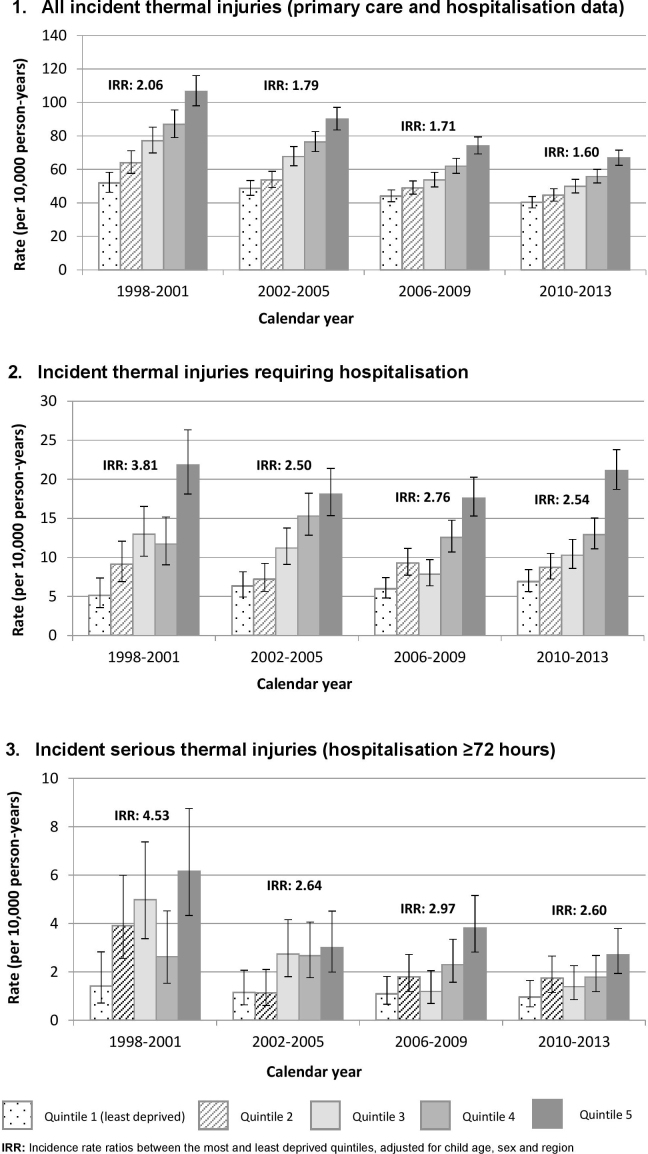
Incidence of thermal injuries in children aged 0–4 according to socioeconomic status and calendar year.

We identified 11,406 thermal injury events among the study cohort, giving a crude incidence rate for the study period of 59.5 per 10,000 PY (Table 2); with incidence rates lower in females compared to males (IRR 0.80, 95%CI 0.77–0.83). Incidence rates peaked among children aged 15–17 months, with a rate of 130.7 per 10,000 PY (95%CI 123.9–138.0) (Supplementary file 3). Incidence rates ranged from 44.9 (95%CI 43.0–47.0) in the least deprived quintile to 79.5 (95%CI 76.6–82.4) in the most deprived quintile. Over the study period, thermal injury incidence fell from 81.3 per 10,000 PY in 1998/99 to 50.0 per 10,000 PY in 2012/13 (IRR 0.64, 95%CI 0.58–0.70) (Fig. 1). Socioeconomic inequalities narrowed over the study period (*p* = 0.004 test for interaction), with children from the most deprived quintiles having a 60% higher rate of thermal injuries than those in the least deprived quintile in the period 2010–2013, compared to a two-fold increased risk in 1998–2001 (Fig. 2).

### 3.2 Thermal injuries undergoing hospitalisation

Amongst the study cohort, 2170 thermal injuries led to hospitalisation during the study period, of which 413 (19.0%) were admitted for 72 h or more (serious thermal injuries). Patterns by child age and gender were similar to overall incidence (Table 2). The socioeconomic gradient between the most and least deprived quintiles was steeper for thermal injuries undergoing hospitalisation (IRR 2.74, 95%CI 2.35–3.20) and serious thermal injuries (IRR 3.17, 95%CI 2.53–3.96), compared to all thermal injuries (IRR 1.75, 95%CI 1.64–1.87). For thermal injuries leading to hospitalisation, incidence rates did not significantly change over time. The incidence rate reduced until 2008/9 (9.8/10,000), after which there was a non-significant increase to 12.7/10,000 PY by 2012/13 (Fig. 1). In contrast, the rate of serious thermal injury significantly reduced across the study period; 56% lower in 2012/13 compared to 1998/9 (IRR 0.44, 95%CI 0.33–0.59). Over time, there was a narrowing in socioeconomic inequalities for injuries undergoing hospitalisation (*p* = 0.01 test for interaction), with children from the most deprived quintiles having a 2.5 time higher rate than those in the least deprived quintile in the period 2010–2013 (IRR 2.54, 95%CI 1.99–3.25), compared to a nearly four-fold increased risk in 1998–2001 (IRR 3.81, 95%CI 2.50–5.80) (Fig. 2). The socioeconomic gradient between the most and least deprived quintiles was narrower in 2010–2013 (IRR 2.60, 95%CI 1.39–4.85) compared to 1998–2001 (IRR 4.53, 95%CI 2.31–8.87) for serious thermal injuries, although not statistically significant (test for interaction *p* = 0.17).

### 3.3 Estimating the burden of thermal injuries

Based upon our estimated thermal injury incidence rates from CPRD and HES data, and the mid-year population estimate for 2013, we estimated that 17,854 thermal injuries occurred among 0–4 year olds in England in 2013, with 4716 events undergoing hospitalisation. For the period 1998–2002, we estimated that 131,826 thermal injuries occurred among 0–4 year olds in the UK.

## 4 Discussion

This study presents detailed data on the incidence of thermal injuries among a cohort of children aged 0–4 years living in England through the use of linked primary care and hospitalisation data. Thermal injury incidence rates were higher among males, and peaked at the age of 15–17 months. Children from the most deprived quintiles had the highest thermal injury rates, with the socioeconomic gradient steepest for serious thermal injuries. Over time there was a narrowing of socioeconomic inequalities between the most and least deprived quintiles for all thermal injuries and thermal injuries undergoing hospitalisation. Over the study period, there was a 36% reduction in the incidence of thermal injuries identified in CPRD and/or HES, and a 56% reduction in serious thermal injuries (admitted for ≥72 h), but no significant change in the incidence of thermal injuries undergoing hospitalisation.

### 4.1 Strengths and limitations

The main strengths of our study are the large study size and use of linked primary care and hospitalisation data. With thermal injuries seen in a range of health care settings, the use of linked health data enables the capture of not only injuries leading to hospitalisation but also more minor thermal injuries (e.g. seen in primary care, seen in ED or minor injury units and recorded in the CPRD), enabling a more complete estimate of thermal injury incidence. While data held in the CPRD are broadly representative of the demographics of the UK population [6], there is some underrepresentation of practices from the North East, East Midlands and Yorkshire and The Humber. As child injury rates are higher in these regions [15], this may lead to some underestimation of injury incidence in our study. Additionally, we have not presented data by region as numbers of practices in some regions are small and so may not be representative of other practices in that region (e.g. practice size, urban/rural location, ethnicity) and as such could be misleading. With future plans for widespread access to primary care data across England, and as CPRD continues to recruit practices, these issues can be resolved, allowing more comprehensive data on injury rates by geographical region.

Through the use of routinely collected health data we are limited by the availability and quality of data recorded. We used admissions lasting 72 h or more as a proxy for serious thermal injuries, as the Read and ICD-10 codes used in CPRD and HES do not allow comprehensive assessment of the extent and severity of burns; key information of interest to those working within burns services. Data on mechanism (e.g. bath water scald) and place of occurrence (e.g. home) are poorly recorded within UK primary care data [11], and as most thermal injuries do not lead to hospitalisation, we have been unable to present comprehensive data on injury mechanisms.

ED data are yet to be linked to the CPRD; an important limitation of our study. GPs receive information about their patients’ attendances at EDs, outpatient clinics and hospitalisations. At present, without linked ED data we are relying upon GPs both receiving information about ED attendances and recording this information in the primary care record. The extent to which ED attendances are captured in the primary care record is unknown and difficult to quantify. While not directly comparable (as will not capture primary care attendances but will capture repeat ED attendances for the same injury), data from the HASS/LASS injury surveillance system estimated that for the period 1998–2002 there were 164,153 burns among 0–4 year olds [14], compared to our estimate of 131,826; indicating an underestimation of at least 20% using our data. While it is likely we have underestimated thermal injury incidence using CPRD and HES, it is unlikely that our observed patterns (e.g. over time, by child age and gender) are affected, as there is little evidence to suggest changes in the capture of injuries seen in ED in the primary care record either over time or according to child characteristics. Future linkage of ED data (when it becomes available) to the CPRD will address this limitation.

### 4.2 Comparison with existing literature

Existing data on the epidemiology of burns most commonly comes from single-centres [16] or analyses of hospitalisation [3], [17] or specialist burn unit datasets [4], [18]. There are no directly comparable data to our estimates of thermal injury incidence using CPRD and HES. Existing injury surveillance systems from high-income countries estimate burn rates as between 21.0 and 31.6/10,000 among 0–4 year olds [19], [20], [21]; lower than our estimate of 59.5/10,000 person-years, in part reflecting our inclusion of thermal injuries seen in primary care. Our rate of thermal injuries undergoing hospitalisation (11.3/10,000) was consistent with data from a study using HES data for all 0–4 year olds from England [3], but higher than rates reported in other high-income countries (3.5–9/10,000) [17], [20], [21], [22], [23]. Differences in hospitalisation rates may reflect differences in admission thresholds and configuration of burn services between countries. Two previous studies from England using hospitalisation [3] and specialist burns unit data [4] observed an increase in hospitalisation rates after 2006–2008; potentially reflecting changes in burns services following the publication of a National Burns Care Review in 2001 [24], and subsequent guidelines on referrals to burns services [25]. Similarly we demonstrated an increase in hospitalisation rates after 2008; but this was not statistically significant in our dataset, potentially due to our smaller number of cases compared to these previous studies focusing on both adults and children from England [3].

Reviews of the epidemiology of burns both in Europe and globally suggest reductions in burn occurrence and severity in high-income countries [26], [27]. In 2014 Dokter et al. demonstrated reductions in both burn severity and length of stay between 1995 and 2011 amongst patients admitted to burn centres in the Netherlands [18]. While our finding of a reduction in the incidence of serious burns during the study period may reflect a true reduction in incidence, by using admission length as a proxy for severity, the observed reduction could also reflect changes in burns management (e.g. shorter admissions due to new treatments or increased availability of outpatient services). Verifying this trend and whether there have been changes in admission thresholds should be assessed in other data sources, such as the International Burn Injury Database [4], that capture detailed data on injury severity.

The observed peak in burns incidence among children aged 15–17 months, with incidence higher among males than females is consistent with existing literature [14], [22]. Across both high and low income countries there is evidence for socioeconomic inequalities in thermal injury occurrences [28], with particularly steep socioeconomic gradients seen for deaths from fires [29]. We observed increasingly steep socioeconomic gradients for thermal injuries undergoing hospitalisation and serious thermal injuries, compared to overall thermal injury incidence. This potentially indicates that those from the most deprived groups not only have higher thermal injury rates, but also more severe thermal injuries; consistent with evidence from some previous injury studies [30], [31]. This finding should however be confirmed in other data sources better able to assess burns severity, as it is possible that some of the elevated risk could be explained by social factors affecting hospitalisation practices (e.g. safeguarding concerns, travel distance).

### 4.3 Conclusions and implications

Using our estimated incidence rates, at least 17,854 thermal injuries occurred among 0–4 year olds in the England in 2013, with 4716 events undergoing hospitalisation. This is likely to be an underestimation, as by using linked primary care and hospitalisation data, we will have only captured ED attendances that were recorded in the primary care record. While our findings show a reduction in the incidence of all thermal injuries (identified in CPRD and/or HES) and serious thermal injuries, this study highlights steep socioeconomic gradients in injury risk, particularly for more severe thermal injuries. In accordance with guidance from the National Institute for Health and Care Excellence [32], preventative interventions should be targeted to the most deprived households. With commissioning responsibilities for services such as health visiting and the Family Nurse Partnership recently transferred to public health teams (from October 2015), these teams, in collaboration with specialised burns services and other agencies (e.g. fire services), need to ensure the delivery of injury prevention strategies and campaigns (e.g. National Burn Awareness Day), and that evidence-based interventions (e.g. home safety schemes and equipment [33]) are in place.

## Conflict of interest

We declare that we have no competing interests.

## Authors’ contributions

DK, EO, LT, TB and RB were involved in the conception and design of the study. RB undertook the analysis and drafted the manuscript. All authors (DK, EO, RB, LT, TB, MK) contributed to interpreting the findings and revising the manuscript.

## Funding

This paper presents independent research funded by the National Institute for Health Research School for Primary Care Research (NIHR SPCR) and The University of Nottingham. The funder had no involvement in the collection, analysis or interpretation of the data. The views expressed are those of the authors and not necessarily those of the NHS, the NIHR or the Department of Health.

## Ethics statement

Approval for this study was obtained from the Independent Scientific Advisory Committee for the Medicines and Healthcare products Regulatory Agency in December 2013.

## Figures and Tables

**Table 1:** Characteristics of the study population, 0–4 year old children living in England with linked CPRD-HES data for the period 1998–2013.

	Frequency (%)
Gender	
Male	369,513 (52.2)
Female	338,537 (47.8)

Age at start of follow-up (years)	
0	489,226 (69.1)
1	65,707 (9.3)
2	57,250 (8.1)
3	51,011 (7.2)
4	44,856 (6.3)

Year of birth	
1993–2000	159,862 (22.6)
2001–2006	237,042 (33.5)
2006–2013	311,146 (43.9)

Socioeconomic status (index of multiple deprivation, 2010)	
Quintile 1 (least deprived)	147,035 (20.8)
Quintile 2	140,433 (19.8)
Quintile 3	132,195 (18.7)
Quintile 4	144,777 (20.4)
Quintile 5 (most deprived)	142,431 (20.1)
Missing	1179 (0.2)

**Table 2:** Crude incidence rates and adjusted incidence rate ratios for all thermal injuries, thermal injuries undergoing hospitalisation, and those hospitalised for 72 h or more, children aged 0–4 from England, 1998–2013 (*n* = 708,050).

		All incident thermal injuries^a^			Incident thermal injuries leading to hospitalisation^b^			Serious thermal injuries, hospitalised ≥72 h^b^, ^c^		
	Person-years	Number of thermal events	Crude incidence rate, per 10,000 person-years (95%CI)	Adjusted IRR (95%CI)^d^	Number of thermal events	Crude incidence rate, per 10,000 person-years (95%CI)	Adjusted IRR (95%CI)^d^	Number of thermal events	Crude incidence rate, per 10,000 person-years (95%CI)	Adjusted IRR (95%CI)^d^
Overall rate	1,917,686	11,406	59.5 (58.4–60.6)	–	2170	11.3 (10.8–11.8)	–	413	2.15 (1.95–2.37)	–

Gender										
Male	1,001,532	6593	65.8 (64.3–67.4)	1	1266	12.6 (12.0–13.4)	1	238	2.38 (2.08–2.70)	1
Female	916,381	4813	52.5 (51.1–54.0)	0.80 (0.77–0.83)	904	9.9 (9.2–10.5)	0.78 (0.71–0.85)	175	1.91 (1.64–2.22)	0.81 (0.71–0.92)

Age at thermal injury (years)										
0	384,358	2287	59.5 (57.1–62.0)	1	447	11.6 (10.6–12.8)	1	76	1.97 (1.58–2.48)	1
1	408,582	4587	112.3 (109.0–115.7)	2.50 (2.30–2.73)	987	24.2 (22.7–25.7)	2.63 (2.19–3.16)	187	4.58 (3.97–5.28)	2.75 (1.99–3.80)
2	391,557	2274	58.1 (55.7–60.6)	2.24 (2.05–2.44)	396	10.1 (9.2–11.2)	2.26 (1.87–2.72)	79	2.02 (1.62–2.52)	2.45 (1.77–3.38)
3	374,313	1322	35.3 (33.5–37.3)	1.16 (1.06–1.28)	191	5.1 (4.4–5.9)	0.98 (0.80–1.21)	37	0.99 (0.72–1.36)	1.02 (0.71–1.45)
4	357,464	936	26.2 (24.5–28.0)	0.75 (0.68–0.83)	149	4.2 (3.5–4.9)	0.57 (0.46–0.71)	34	0.95 (0.68–1.33)	0.76 (0.53–1.08)

IMD quintile										
1 (least deprived)	426,517	1917	44.9 (43.0–47.0)	1	266	6.2 (5.5–7.0)	1	47	1.10 (0.81–1.47)	1
2	391,483	1986	50.7 (48.5–53.0)	1.15 (1.07–1.23)	337	8.6 (7.7–9.6)	1.34 (1.13–1.59)	75	1.92 (1.51–2.40)	1.75 (1.38–2.23)
3	355,715	2096	58.9 (56.4–61.5)	1.32 (1.24–1.41)	360	10.1 (9.1–11.2)	1.56 (1.32–1.85)	76	2.14 (1.68–2.67)	1.92 (1.50–2.47)
4	378,844	2510	66.3 (63.7–68.9)	1.51 (1.41–1.61)	499	13.2 (12.0–14.4)	2.00 (1.70–2.36)	85	2.24 (1.79–2.77)	2.03 (1.61–2.56)
5	362,842	2884	79.5 (76.6–82.4)	1.75 (1.64–1.87)	708	19.5 (18.1–21.0)	2.74 (2.35–3.20)	130	3.58 (2.99–4.25)	3.17 (2.53–3.96)
Missing	2284	13	56.9 (30.3–97.3)	1.25 (0.73–2.12)	0	–	–	0	–	–

Calendar year										
1998–1999	103,403	841	81.3 (75.9–87.0)	1	124	12.0 (10.0–12.3)	1	38	3.67 (2.60–5.04)	1
2000–2001	157,327	1152	73.2 (69.0–77.6)	0.92 (0.84–1.01)	187	11.9 (10.2–13.7)	1.02 (0.80–1.29)	60	3.81 (2.91–4.91)	1.07 (0.81–1.41)
2002–2003	192,774	1286	66.7 (63.1–70.5)	0.87 (0.79–0.95)	221	11.5 (10.0–13.1)	1.01 (0.80–1.29)	40	2.07 (1.48–2.83)	0.60 (0.44–0.81)
2004–2005	231,956	1526	65.8 (62.5–69.2)	0.87 (0.79–0.95)	259	11.2 (9.8–12.6)	0.94 (0.75–1.18)	48	2.07 (1.53–2.74)	0.56 (0.42–0.75)
2006–2007	282,787	1683	59.5 (56.7–62.4)	0.82 (0.74–0.89)	316	11.2 (10.0–12.5)	0.95 (0.77–1.19)	62	2.19 (1.68–2.81)	0.60 (0.46–0.79)
2008–2009	315,580	1665	52.8 (50.3–55.4)	0.74 (0.68–0.81)	308	9.8 (8.7–10.9)	0.85 (0.68–1.06)	57	1.81 (1.37–2.34)	0.51 (0.38–0.68)
2010–2011	323,577	1702	52.6 (50.1–55.2)	0.67 (0.62–0.73)	360	11.1 (10.0–12.3)	0.96 (0.77–1.19)	58	1.79 (1.36–2.32)	0.50 (0.38–0.66)
2012–2013	310,278	1551	50.0 (47.5–52.5)	0.64 (0.58–0.70)	395	12.7 (11.5–14.1)	1.08 (0.87–1.34)	50	1.61 (1.20–2.13)	0.44 (0.33–0.59)

a Incident thermal injuries identified from linked primary care and hospitalisation data using a time-based algorithm to remove repeat records for the same injury event.

b Incident hospitalisations identified from Hospital Episode Statistics with readmissions for the same injury event excluded.

c Admission for 72 h or more was used as a proxy for serious thermal injuries.

d mutually adjusted for child age, gender, socioeconomic status, calendar time and region.
