# Supplementary material for: Differing patterns in thermal injury incidence and hospitalisations among 0–4 year old children from England
Source: Burns. 2016 Nov;42(7):1609–16. doi: 10.1016/j.burns.2016.05.007 (PMC5062947; doi:10.1016/j.burns.2016.05.007)
Supplement: Supplementary file 1 [file mmc1.pdf]

Supplementary file 1: List of Read codes referring to thermal injuries

- Read codes are used to extract thermal injury records from the CPRD (primary care data).

**Read Code**	**Description of Read code**
7G2C.00	Operations on burnt skin
7G2C.11	Operations on burnt skin including head or neck
7G2C100	Toilet or clean burnt skin NEC
7G2C200	Debridement of burnt skin of head and neck
7G2C300	Debridement of burnt skin NEC
7G2C500	Tangent excision of burnt skin of head or neck
7G2C600	Tangent excision of burnt skin NEC
7G2C700	Escharotomy of burnt skin of head
7G2C900	Escharotomy of burnt skin of chest
7G2CA00	Escharotomy of burnt skin of arm
7G2CB00	Escharotomy of burnt skin of hand
7G2CC00	Escharotomy of burnt skin of leg
7G2CD00	Escharotomy of burnt skin of foot
7G2CE00	Removal of slough from burnt skin NEC
7G2CE11	Escharotomy of burnt skin NEC
7G2CG00	Dress burnt skin head or neck us vacuum assisted clos device
7G2CH00	Cleansing and sterilisation of burnt skin NEC
7G2Cy00	Other specified toilet to burnt skin
7G2Cz00	Toilet to burnt skin NOS
7G2E000	Dressing of burnt skin of head or neck
7G2E100	Dressing of burnt skin NEC
7G2E600	Attention to dressing of burn of head or neck
7G2E700	Attention to dressing of burnt skin NEC
7G2F.11	Exploration of skin wound or burn
7G2F300	Exploration of burnt skin NEC
7G2Fv00	Exploration of burnt skin of other site NOS
81H2.00	Dressing of burn
8H15.00	Admit to burns unit
8H5E.00	Burns referral
9N0z.00	Seen in burns clinic
9b8A000	Burns care
R020100	[D]Burning of skin
SH...00	Burns
SH...11	Scalds
SH0..00	Burn confined to eye and adnexa
SH0..11	Conjunctival burns
SH0..12	Corneal burns
SH0..13	Eyelid burns
SH0..14	Periocular burns
SH01.00	Other burns of eyelids and periocular area
SH05.00	Burn resulting in eyeball rupture and destruction of eyeball
SH0x.00	Burn of eyelid NOS

SH0y.00	Burn of cornea NOS
SH0z.00	Burn confined to eye and adnexa NOS
SH1..00	Burn of the face, head or neck
SH1..11	Face burns
SH1..12	Head burns
SH10.00	Unspecified thickness burn of the face, head or neck
SH10000	Unspecified thickness burn of unspecified part of face/head
SH10100	Unspecified thickness burn of the ear
SH10200	Unspecified thickness burn of the eye
SH10300	Unspecified thickness burn of the lip(s)
SH10400	Unspecified thickness burn of the chin
SH10500	Unspecified thickness burn of the nose
SH10600	Unspecified thickness burn of the scalp
SH10700	Unspecified thickness burn of the forehead
SH10800	Unspecified thickness burn of the cheek
SH10900	Unspecified thickness burn of the neck
SH10x00	Unspecified thickness burn multiple sites face, head or neck
SH10z00	Unspecified thickness burn of the face, head or neck NOS
SH11.00	Superficial burn of the face, head or neck
SH11.11	Erythema of head or neck, first degree burn
SH11000	Superficial burn of unspecified part of the face or head
SH11100	Superficial burn of the ear
SH11200	Superficial burn of the eye
SH11300	Superficial burn of the lip(s)
SH11400	Superficial burn of the chin
SH11500	Superficial burn of the nose
SH11600	Superficial burn of the scalp
SH11700	Superficial burn of the forehead
SH11800	Superficial burn of the cheek
SH11900	Superficial burn of the neck
SH11x00	Superficial burn of multiple sites of the face, head or neck
SH11z00	Superficial burn of the face, head or neck NOS
SH12.00	Partial thickness burn of the face, head or neck
SH12.11	Blister of face, head and neck, second degree burn
SH12000	Superficial part. thickness burn unspecified part face/head
SH12100	Superficial partial thickness burn of the ear
SH12111	Ear - 2nd degree burn
SH12200	Superficial partial thickness burn of the eye
SH12211	Eye - 2nd degree burn
SH12300	Superficial partial thickness burn of the lip(s)
SH12311	Lip - 2nd degree burn
SH12400	Superficial partial thickness burn of the chin
SH12500	Superficial partial thickness burn of the nose
SH12600	Superficial partial thickness burn of the scalp
SH12611	Scalp - 2nd degree burn
SH12700	Superficial partial thickness burn of the forehead

SH12711	Forehead - 2nd degree burn
SH12800	Superficial partial thickness burn of the cheek
SH12811	Cheek - 2nd degree burn
SH12900	Superficial partial thickness burn of the neck
SH12A00	Deep partial thickness burn of unspecified part of face/head
SH12B00	Deep partial thickness burn of the ear
SH12C00	Deep partial thickness burn of the eye
SH12E00	Deep partial thickness burn of the chin
SH12G00	Deep partial thickness burn of the scalp
SH12x00	Partial thickness burn of multiple sites face, head or neck
SH12z00	Partial thickness burn of the face, head or neck NOS
SH13.00	Full thickness burn of the face, head or neck
SH13000	Full thickness burn of unspecified part of the face or head
SH13100	Full thickness burn of the ear
SH13200	Full thickness burn of the eye
SH13300	Full thickness burn of the lip(s)
SH13500	Full thickness burn of the nose
SH13600	Full thickness burn of the scalp
SH13700	Full thickness burn of the forehead
SH13800	Full thickness burn of the cheek
SH13900	Full thickness burn of the neck
SH13x00	Full thickness burn of multiple sites of face, head or neck
SH14100	Deep full thickness burn of ear without loss of body part
SH14200	Deep full thickness burn of eye without loss of body part
SH14600	Deep full thickness burn of scalp without loss of body part
SH14900	Deep full thickness burn of neck without loss of body part
SH15100	Deep full thickness burn of the ear, with loss of body part
SH1z.00	Burn of the face, head or neck NOS
SH2..00	Burn of the trunk
SH20.00	Unspecified thickness burn of the trunk
SH20000	Unspecified thickness burn of unspecified part of the trunk
SH20100	Unspecified thickness burn of the breast
SH20200	Unspecified thickness burn of the chest wall
SH20300	Unspecified thickness burn of the abdominal wall
SH20400	Unspecified thickness burn of the back (excluding buttock)
SH20500	Unspecified thickness burn of the buttock
SH20600	Unspecified thickness burn of the genitalia
SH20x00	Unspecified thickness burn of multiple sites of the trunk
SH20z00	Unspecified thickness burn of the trunk NOS
SH21.00	Superficial burn of the trunk
SH21.11	Erythema of trunk, 1st degree burn
SH21000	Superficial burn of unspecified part of the trunk
SH21100	Superficial burn of the breast
SH21200	Superficial burn of the chest wall
SH21300	Superficial burn of the abdominal wall
SH21400	Superficial burn of the back (excluding buttock)

SH21500	Superficial burn of the buttock
SH21600	Superficial burn of the genitalia
SH21z00	Superficial burn of the trunk NOS
SH22.00	Partial thickness burn of the trunk
SH22.11	Blister of trunk, second degree burn
SH22000	Superficial partial thickness burn unspecified part of trunk
SH22100	Superficial partial thickness burn of the breast
SH22200	Superficial partial thickness burn of the chest wall
SH22300	Superficial partial thickness burn of the abdominal wall
SH22400	Superficial partial thickness burn of back (excl buttock)
SH22500	Superficial partial thickness burn of the buttock
SH22600	Superficial partial thickness burn of the genitalia
SH22800	Deep partial thickness burn of the breast
SH22900	Deep partial thickness burn of the chest wall
SH22A00	Deep partial thickness burn of the abdominal wall
SH22B00	Deep partial thickness burn of the back (excluding buttock)
SH22C00	Deep partial thickness burn of the buttock
SH22x00	Partial thickness burn of multiple sites of the trunk
SH22z00	Partial thickness burn of the trunk NOS
SH23.00	Full thickness burn of the trunk
SH23000	Full thickness burn of the trunk, unspecified
SH23100	Full thickness burn of the breast
SH23200	Full thickness burn of the chest wall
SH23300	Full thickness burn of the abdominal wall
SH23400	Full thickness burn of the back (excluding buttock)
SH23500	Full thickness burn of the buttock
SH23z00	Full thickness burn of the trunk NOS
SH24.00	Deep full thickness burn of trunk without loss of body part
SH24000	Deep full thickness burn of trunk unsp, no loss of body part
SH24100	Deep full thickness burn of breast without loss of body part
SH24200	Deep full thickness burn of chest without loss of body part
SH24300	Deep full thickness burn of abdom.wall, no loss of body part
SH24400	Deep full thickness burn of back without loss of body part
SH24500	Deep full thickness burn of buttock, no loss of body part
SH25.00	Deep full thickness burn of trunk, with loss of body part
SH25200	Deep full thickness burn of chest, with loss of body part
SH25500	Deep full thickness burn of buttock, with loss of body part
SH2z.00	Burn of the trunk NOS
SH3..00	Burn of the arm (excluding wrist and hand)
SH30.00	Unspecified thickness burn of the arm
SH30000	Unspecified thickness burn of the arm, unspecified
SH30100	Unspecified thickness burn of the forearm
SH30200	Unspecified thickness burn of the elbow
SH30300	Unspecified thickness burn of the upper arm
SH30400	Unspecified thickness burn of the axilla
SH30500	Unspecified thickness burn of the shoulder

SH30600	Unspecified thickness burn of the scapular region
SH30x00	Unspecified thickness burn of multiple sites of the arm
SH30z00	Unspecified thickness burn of the arm NOS
SH31.00	Superficial burn of the arm
SH31.11	Erythema of arm, first degree burn
SH31000	Superficial burn of the arm, unspecified
SH31100	Superficial burn of the forearm
SH31200	Superficial burn of the elbow
SH31300	Superficial burn of the upper arm
SH31400	Superficial burn of the axilla
SH31500	Superficial burn of the shoulder
SH31600	Superficial burn of the scapular region
SH31x00	Superficial burn of multiple sites of the arm
SH31z00	Superficial burn of the arm NOS
SH32.00	Partial thickness burn of the arm
SH32.11	Blister of arm, second degree burn
SH32000	Superficial partial thickness burn of the arm, unspecified
SH32100	Superficial partial thickness burn of the forearm
SH32200	Superficial partial thickness burn of the elbow
SH32300	Superficial partial thickness burn of the upper arm
SH32500	Superficial partial thickness burn of the shoulder
SH32600	Superficial partial thickness burn of scapular region
SH32700	Deep partial thickness burn of the arm, unspecified
SH32800	Deep partial thickness burn of the forearm
SH32900	Deep partial thickness burn of the elbow
SH32A00	Deep partial thickness burn of the upper arm
SH32D00	Deep partial thickness burn of the scapular region
SH32x00	Partial thickness burn of multiple sites of the arm
SH32z00	Partial thickness burn of the arm NOS
SH33.00	Full thickness burn of the arm
SH33000	Full thickness burn of the arm, unspecified
SH33100	Full thickness burn of the forearm
SH33200	Full thickness burn of the elbow
SH33300	Full thickness burn of the upper arm
SH33400	Full thickness burn of the axilla
SH33500	Full thickness burn of the shoulder
SH34.00	Deep full thickness burn of arm without loss of body part
SH34100	Deep full thickness burn of forearm, no loss of body part
SH34200	Deep full thickness burn of elbow without loss of body part
SH34300	Deep full thickness burn of upper arm, no loss of body part
SH34500	Deep full thickness burn of shoulder, no loss of body part
SH35400	Deep full thickness burn of axilla, with loss of body part
SH3z.00	Burn of the arm (excluding wrist and hand) NOS
SH4..00	Burn of the wrist(s) and hand(s)
SH40.00	Unspecified thickness burn of the wrist and hand
SH40.11	Unspecified degree burn of finger

SH40.12	Unspecified degree burn of hand
SH40.13	Unspecified degree burn of thumb
SH40.14	Unspecified degree burn of wrist
SH40000	Unspecified thickness burn of the hand, unspecified
SH40100	Unspecified thickness burn of a single finger
SH40200	Unspecified thickness burn of the thumb
SH40300	Unspecified thickness burn of more than one finger
SH40400	Unspecified thickness burn of the thumb and finger(s)
SH40500	Unspecified thickness burn of the palm of hand
SH40600	Unspecified thickness burn of the back of hand
SH40700	Unspecified thickness burn of the wrist
SH40x00	Unspecified thickness burn of multiple sites of wrist/hand
SH40z00	Unspecified thickness burn of the wrist or hand NOS
SH41.00	Superficial burn of the wrist and hand
SH41.11	Erythema of wrist and hand,first degree burn
SH41.12	First degree burn of finger
SH41.13	First degree burn of hand
SH41.14	First degree burn of thumb
SH41.15	First degree burn of wrist
SH41000	Superficial burn of the hand, unspecified
SH41100	Superficial burn of a single finger
SH41200	Superficial burn of the thumb
SH41300	Superficial burn of more than one finger
SH41400	Superficial burn of the thumb and finger(s)
SH41500	Superficial burn of the palm of hand
SH41600	Superficial burn of the back of hand
SH41700	Superficial burn of the wrist
SH41x00	First degree burn of multiple sites of the wrist or hand
SH41z00	Superficial burn of the wrist or hand NOS
SH42.00	Partial thickness burn of the wrist and hand
SH42.11	Blister of wrist and hand, second degree burn
SH42.12	Second degree burn of finger
SH42.13	Second degree burn of hand
SH42.14	Second degree burn of thumb
SH42.15	Second degree burn of wrist
SH42000	Superficial partial thickness burn of hand, unspecified
SH42100	Superficial partial thickness burn of a single finger
SH42200	Superficial partial thickness burn of the thumb
SH42300	Superficial partial thickness burn of more than one finger
SH42400	Superficial partial thickness burn of thumb and finger(s)
SH42500	Superficial partial thickness burn of palm of hand
SH42600	Superficial partial thickness burn of back of hand
SH42700	Superficial partial thickness burn of the wrist
SH42800	Deep partial thickness burn of the hand, unspecified
SH42900	Deep partial thickness burn of a single finger
SH42A00	Deep partial thickness burn of the thumb

SH42D00	Deep partial thickness burn of the palm of hand
SH42E00	Deep partial thickness burn of back of hand
SH42F00	Deep partial thickness burn of wrist
SH42x00	Partial thickness burn of multiple sites of the wrist/hand
SH42z00	Partial thickness burn of the wrist or hand NOS
SH43.00	Full thickness burn of the wrist and hand
SH43.11	Third degree burn of finger
SH43.12	Third degree burn of hand
SH43.14	Third degree burn of wrist
SH43000	Full thickness burn of the hand, unspecified
SH43100	Full thickness burn of a single finger
SH43200	Full thickness burn of the thumb
SH43300	Full thickness burn of more than one finger
SH43400	Full thickness burn of the thumb and finger(s)
SH43500	Full thickness burn of the palm of hand
SH43600	Full thickness burn of the back of hand
SH43700	Full thickness burn of the wrist
SH43z00	Full thickness burn of the wrist or hand NOS
SH44.00	Deep full thickness burn of wrist/hand, no loss of body part
SH44.11	Deep third degree burn of finger,without loss of a body part
SH44.12	Deep third degree burn of hand, without loss of a body part
SH44.13	Deep third degree burn of thumb, without loss of a body part
SH44.14	Deep third degree burn of wrist, without loss of a body part
SH44000	Deep full thickness burn of hand unsp, no loss of body part
SH44100	Deep full thickness burn of a finger, no loss of body part
SH44200	Deep full thickness burn of thumb without loss of body part
SH44400	Deep full thickness burn of thumb+fing, no loss of body part
SH44500	Deep full thickness burn of palm hand, no loss of body part
SH44700	Deep full thickness burn of wrist without loss of body part
SH45.11	Deep third degree burn of finger with loss of a body part
SH45.14	Deep third degree burn of wrist with loss of a body part
SH4z.00	Burn of wrist or hand NOS
SH5..00	Burn of lower limbs
SH5..11	Leg burns
SH50.00	Unspecified thickness burn of the leg
SH50000	Unspecified degree burn of the leg, unspecified
SH50100	Unspecified thickness burn of the toe(s)
SH50200	Unspecified thickness burn of the foot
SH50300	Unspecified thickness burn of the ankle
SH50400	Unspecified thickness burn of the lower leg
SH50500	Unspecified thickness burn of the knee
SH50600	Unspecified thickness burn of the thigh
SH50x00	Unspecified thickness burn of multiple sites of the leg
SH50z00	Unspecified thickness burn of the leg NOS
SH51.00	Superficial burn of the leg
SH51.11	Erythema of leg, first degree burn

SH51000	Superficial burn of the leg, unspecified
SH51100	Superficial burn of the toe(s)
SH51200	Superficial burn of the foot
SH51300	Superficial burn of the ankle
SH51400	Superficial burn of the lower leg
SH51500	Superficial burn of the knee
SH51600	Superficial burn of the thigh
SH51x00	Superficial burn of multiple sites of the leg
SH51z00	Superficial burn of the leg NOS
SH52.00	Partial thickness burn of the leg
SH52.11	Blister of leg, second degree burn
SH52000	Superficial partial thickness burn of the leg, unspecified
SH52100	Superficial partial thickness burn of the toe(s)
SH52200	Superficial partial thickness burn of the foot
SH52300	Superficial partial thickness burn of the ankle
SH52400	Superficial partial thickness burn of the lower leg
SH52500	Superficial partial thickness burn of the knee
SH52600	Superficial partial thickness burn of the thigh
SH52700	Deep partial thickness burn of the leg, unspecified
SH52800	Deep partial thickness burn of the toe(s)
SH52900	Deep partial thickness burn of the foot
SH52A00	Deep partial thickness burn of the ankle
SH52B00	Deep partial thickness burn of the lower leg
SH52C00	Deep partial thickness burn of the knee
SH52D00	Deep partial thickness burn of the thigh
SH52x00	Partial thickness burn of multiple sites of the leg
SH52z00	Partial thickness burn of the leg NOS
SH53.00	Full thickness burn of the leg
SH53000	Full thickness burn of the leg, unspecified
SH53100	Full thickness burn of the toe(s)
SH53200	Full thickness burn of the foot
SH53300	Full thickness burn of the ankle
SH53400	Full thickness burn of the lower leg
SH53500	Full thickness burn of the knee
SH53600	Full thickness burn of the thigh
SH53x00	Full thickness burn of multiple sites of the leg
SH53z00	Full thickness burn of the leg NOS
SH54.00	Deep full thickness burn of leg without loss of body part
SH54000	Deep full thickness burn of leg unsp, no loss of body part
SH54200	Deep full thickness burn of foot without loss of body part
SH54300	Deep full thickness burn of ankle without loss of body part
SH54400	Deep full thickness burn of lower leg without loss of body
SH54500	Deep full thickness burn of knee without loss of body part
SH54600	Deep full thickness burn of thigh without loss of body part
SH55.00	Deep full thickness burn of leg, with loss of body part
SH55100	Deep full thickness burn of toe(s), with loss of body part

SH55200	Deep full thickness burn of foot, with loss of body part
SH55300	Deep full thickness burn of ankle, with loss of body part
SH5z.00	Burn of the lower limb NOS
SH6..00	Burn of multiple specified sites
SH60.00	Unspecified thickness burn of multiple specified sites
SH61.00	Superficial burn of multiple specified sites
SH62.00	Partial thickness burn of multiple specified sites
SH62000	Superficial partial thickness burn multiple specified sites
SH62100	Deep partial thickness burn of multiple specified sites
SH63.00	Full thickness burn of multiple specified sites
SH7..00	Burn of internal organs
SH70.00	Burn of the mouth and pharynx
SH70000	Burn of the mouth, unspecified
SH70100	Burn of the gum
SH70200	Burn of the tongue
SH70300	Burn of the pharynx
SH70z00	Burn of the mouth or pharynx NOS
SH71.00	Burn of the larynx, trachea and lung
SH71000	Burn of the larynx
SH71100	Burn of the trachea
SH71200	Burn of the lung
SH71z00	Burn of the larynx, trachea or lung NOS
SH72.00	Burn of the oesophagus
SH73.00	Burn of the gastrointestinal tract
SH73000	Burn of the stomach
SH73100	Burn of the small intestine
SH73300	Burn of the rectum
SH73z00	Burn of the gastrointestinal tract NOS
SH74.00	Burn of the vagina and uterus
SH74000	Burn of the vagina
SH74z00	Burn of the vagina or uterus NOS
SH7z.00	Burn of internal organ NOS
SH8..00	Burns as a percentage of body surface (BS) involved
SH80.00	Burn involving <10% of body surface (BS)
SH80000	Burn:<10% of body surface, 10%/unspec BS full thickness
SH80z00	Burn:<10% of body surface NOS
SH81.00	Burn involving 10-19% of body surface (BS)
SH81000	Burn: 10-14% of body surface,<10%/unsp BS full thickness
SH81100	Burn: 10-14% of body surface, 10-14% BS full thickness
SH81z00	Burn: 10-19% of body surface NOS
SH82.00	Burn involving 20-29% of body surface (BS)
SH82000	Burn: 20-29% of body surface,<10%/unspec BS full thickness
SH82z00	Burn: 20-29% of body surface NOS
SH83.00	Burn involving 30-39% of body surface (BS)
SH83z00	Burn: 30-39% of body surface NOS
SH84.00	Burn involving 40-49% of body surface (BS)

SH84400	Burn: 40-49% of body surface, 40-49% BS full thickness
SH84z00	Burn: 40-49% of body surface NOS
SH85.00	Burn involving 50-59% of body surface (BS)
SH85400	Burn: 50-59% of body surface, 40-49% BS full thickness
SH85z00	Burn: 50-59% of body surface NOS
SH86.00	Burn involving 60-69% of body surface (BS)
SH87.00	Burn involving 70-79% of body surface (BS)
SH87500	Burn: 70-79% of body surface, 50-59% BS full thickness
SH89.00	Burn involving >90% of body surface (BS)
SH8z.00	Burn as a percentage of body surface involved NOS
SH9..00	Burn - unspecified
SH90.00	Unspecified degree of burn NOS
SH91.00	Superficial burn NOS
SH91.11	First degree burn
SH92.00	Partial thickness burn NOS
SH92.11	Second degree burn
SH92000	Superficial partial thickness burn NOS
SH92100	Deep partial thickness burn NOS
SH93.00	Full thickness burn NOS
SH93.11	Third degree burn
SH94.00	Deep full thickness burn, without loss of body part, NOS
SH9z.00	Burn - unspecified
SHz..00	Burns NOS
SM7y200	Smoke inhalation
SM7z.11	Smoke inhalation
SyuD.00	[X]Burns and corrosions
SyuD000	[X]Burns of other parts of eye and adnexa
SyuD500	[X]Burns of other and unspecified internal organs
SyuD800	[X]Burns of mult reg, at least 1 burn of 3rd deg mentioned
SyuDA00	[X]Burn of unspecified body region, unspecified degree
T03..00	Train accident involving explosion, fire or burning
T032.00	Train accident involving burning
T338.00	Fire in road vehicle NEC
T410.00	Burned while ship on fire
T504.00	Fire on aircraft while taking off
T504700	Aircraft fire on takeoff - parachutist injured
T514z00	Fire on aircraft-flying - other person injured
TD...00	Accidents caused by fire and flames
TD0..00	Conflagration in private dwelling
TD0..11	House fire
TD00500	Explosion caused by conflagration in house
TD01.00	Fumes from combustion of PVC in conflagration-private dwell
TD02.00	Carbon monoxide fumes from conflagration in private dwelling
TD02000	Carbon monoxide fumes from conflagration in apartment
TD02500	Carbon monoxide fumes from conflagration in house
TD02800	Carbon monoxide fumes from conflagration in private garage

TD02z00	Carbon monoxide fumes from conflagration private dwell NOS
TD03.00	Fumes NOS from conflagration in private dwelling
TD03000	Fumes NOS from conflagration in apartment
TD03500	Fumes NOS from conflagration in house
TD04.00	Smoke NOS from conflagration in private dwelling
TD04500	Smoke NOS from conflagration in house
TD04A00	Smoke NOS from conflagration in tenement
TD04z00	Smoke NOS from conflagration in private dwelling NOS
TD05.00	Burning caused by conflagration in private dwelling
TD05000	Burning caused by conflagration in apartment
TD05200	Burning caused by conflagration in camping place
TD05500	Burning caused by conflagration in house
TD05z00	Burning caused by conflagration in private dwelling NOS
TD06100	Accident due to collapse of burning boarding house
TD07.00	Accident due to fall from burning private dwelling
TD07300	Accident due to fall from burning caravan
TD07500	Accident due to fall from burning house
TD08300	Hit by object falling from burning caravan
TD08400	Hit by object falling from burning farmhouse
TD09.00	Jump from burning private dwelling
TD09z00	Jump from burning private dwelling NOS
TD0z.00	Accidents caused by conflagration in private dwelling NOS
TD1..00	Conflagration in other building or structure
TD10.00	Explosion caused by conflagration - other building/structure
TD11.00	Fumes from combustion of PVC in fire, in other structure
TD12.00	Carbon monoxide fumes from fire in other structure/building
TD12z00	Carbon monoxide fumes from fire in structure or building NOS
TD13.00	Fumes NOS from conflagration in structure or building
TD13500	Fumes NOS from conflagration in factory
TD13z00	Fumes NOS from conflagration in structure or building NOS
TD14.00	Smoke NOS from conflagration in structure or building
TD15.00	Burning caused by conflagration in other structure/building
TD16.00	Accident due to collapse of other burning structure/building
TD17.00	Accident due to fall from other burning structure/building
TD18100	Hit by object falling from burning church
TD19.00	Jump from other burning structure or building
TD19100	Jump from burning church
TD19800	Jump from burning hotel
TD19z00	Jump from burning structure or building NOS
TD1y.00	Other accident due to fire in other structure/building
TD20.00	Uncontrolled fire in forest
TD21.00	Uncontrolled fire in grass
TD22.00	Uncontrolled fire in hay
TD23.00	Uncontrolled lumber fire
TD24.00	Uncontrolled fire in mine
TD25.00	Uncontrolled fire on prairie

TD3..00	Accidents caused by clothes on fire, ACOF
TD30000	ACOF-contr fire in private dwelling - normal charcoal fire
TD30300	ACOF-contr fire in private dwelling - normal gas fire
TD30500	ACOF-contr fire in private dwelling - brazier
TD32200	ACOF-controlled fire in the open due to trash fire
TD3y300	Accident caused by clothes on fire from candle
TD3y400	Accident caused by clothes on fire from cigar
TD3y500	Accident caused by clothes on fire from cigarette
TD3y600	Accident caused by clothes on fire from lighter
TD3y700	Accident caused by clothes on fire from matches
TD3y900	Accident caused by clothes on fire from welding torch
TD3z.00	Ignition of clothing NOS
TD41.00	Ignition of gasoline with ignition of clothing
TD42.00	Ignition of fat with ignition of clothing
TD44.00	Ignition of paraffin with ignition of clothing
TD45.00	Ignition of petrol with ignition of clothing
TD46.00	Ignition of liquid paraffin gas with ignition of clothing
TD4z.00	Ignition of highly inflammable material NOS
TD5..00	Accident caused by controlled fire in private dwelling
TD52.00	Accident caused by normal electric fire in private dwelling
TD53.00	Accident caused by normal gas fire in private dwelling
TD57.00	Accident caused by cooker, unspecified, in private dwelling
TD57000	Accident caused by gas cooker in private dwelling
TD57200	Accident caused by electric cooker in private dwelling
TD57300	Accident by liquid paraffin gas cooker in private dwelling
TD5z.00	Accident caused by fireplace in private dwelling NOS
TD62.00	Accident caused by normal electric fire other struct/build
TD63.00	Accident caused by normal gas fire other structure/building
TD66.00	Accident caused by furnace in other structure or building
TD70.00	Accident caused by controlled fire in the open, bonfire
TD72.00	Accident caused by controlled fire in the open, trash fire
TDy..00	Accident caused by other fire and flames
TDy0.00	Burning bedclothes
TDyy.00	Accident caused by other flame
TDyy200	Accident caused by candle
TDyy300	Accident caused by cigar
TDyy400	Accident caused by cigarette
TDyy500	Accident caused by lamp
TDyy600	Accident caused by lighter
TDyy800	Accident caused by pipe
TDyy900	Accident caused by welding torch
TDyz.00	Accident caused by other flame or fire NOS
TDz..00	Accident caused by fire or flames NOS
TDz1.00	Accident caused by unspecified fire
TG30000	Accidentally burned by machinery
TG70.00	Accident caused by fireworks

TG8..00	Accidents caused by hot substance, caustic/corrosive, steam
TG80.00	Accidents caused by hot liquids and vapours,including steam
TG80000	Accidental burning/scalding caused by boiling water, unspec
TG80100	Accidental burning/scalding caused by boiling liquid, unspec
TG80200	Accidental burning or scalding caused by liquid metal
TG80300	Accidental burning or scalding caused by steam
TG80400	Accidental burning/scalding by boiling water from kettle
TG80500	Accidental burning/scalding by boiling water from saucepan
TG80600	Accidental burning or scalding caused by tea
TG80700	Accidental burning or scalding caused by coffee
TG80800	Accidental burning or scalding caused by chocolate
TG80900	Accidental burning or scalding caused by milk
TG80A00	Accidental burning/scalding caused by soup, stew or curries
TG80B00	Accidental burning or scalding caused by fat
TG80C00	Accidental burning or scalding caused by steam from kettle
TG80D00	Accidental burning or scalding by steam from car radiator
TG80y00	Accidental burning or scalding caused by other hot vapour
TG80z00	Accidental burning/scalding caused by hot liquid/vapour NOS
TG8y.00	Accidental burning caused by other hot substance or object
TG8y000	Accid burning caused by heat from electric heating appliance
TG8y100	Accidental burning caused by light bulb
TG8y200	Accidental burning caused by steam pipe
TG8y300	Accidental burning or scalding caused by bitumen or tar
TG8y400	Accidental burning or scalding caused by plastic
TG8yz00	Accidental burning caused by hot object NOS
TG8z.00	Accident caused by hot substance,caustic/corrosive,steam NOS
TG9z000	Accidental burn or other injury from electric current NOS
TN81.00	Injury ?accidental, by burns or fire
TN82.00	Injury ?accidental, by scald
U16..00	[X]Exposure to smoke, fire and flames
U160000	[X]Exposure to uncontr fire in building/structur occ home
U161000	[X]Exposur uncontrol fire not in building/structure occ home
U162000	[X]Exposure to controlld fire in building/structur occ home
U163.00	[X]Exposure to controlled fire, not in building / structure
U163011	[X]Exposure to bonfire
U16y.00	[X]Exposure to other specified smoke, fire and flames
U16y000	[X]Exposure to oth specif smoke fire+flames occurrn at home
U16z.00	[X]Exposure to unspecified smoke, fire and flames
U16z000	[X]Exposure to unspecifd smoke fire/flames occurrn at home
U16z200	[X]Exposr unspecif smoke fire/flame sch oth ins/pub adm area
U16z600	[X]Exposur unspecif smoke fire/flame occ indust/constr area
U16zz00	[X]Exposur unspecif smoke fire/flame occurrn unspecif place
U17..00	[X]Contact with heat and hot substances
U17..11	[X]Cause of accident burn / scald
U170.00	[X]Contact with hot drinks, food, fats and cooking oils
U170000	[X]Contact with hot drink food fat+cooking oil occurrn home

U170200	[X]Cont hot drink food fat+cook oil sch oth ins/pub adm area
U170z00	[X]Contact hot drink food fat+cook oil occurrn unspecif plce
U171.00	[X]Contact with hot tap-water
U171000	[X]Contact with hot tap-water, occurrence at home
U172.00	[X]Contact with other hot fluids
U172000	[X]Contact with other hot fluids, occurrence at home
U172z00	[X]Contact with other hot fluids occurrn at unspecif place
U173.00	[X]Contact with steam and hot vapours
U173000	[X]Contact with steam and hot vapours, occurrence at home
U173200	[X]Contact with steam+hot vapour, sch oth inst/pub adm area
U173500	[X]Contact with steam+hot vapour occurrn trade/service area
U174.00	[X]Contact with hot air and gases
U175.00	[X]Contact with hot household appliances
U175000	[X]Contact with hot household appliances occurrence at home
U175600	[X]Contact wth hot househld applianc occ indust/constr area
U176.00	[X]Contact with hot heating appliances, radiators and pipes
U177000	[X]Contact with hot engines machinery+tools occurrn at home
U177500	[X]Contct wth hot engin machinry+tool occ trade/servce area
U178.00	[X]Contact with other hot metals
U178z00	[X]Contact with other hot metals occurrn at unspecif place
U17y.00	[X]Contact with other and unspecif heat and hot substances
U17y000	[X]Contact with oth+unspecif heat+hot substnc occurrn home
U17y600	[X]Contact oth+unspec heat+hot subst occ indust/constr area
U17yz00	[X]Contact oth+unspecif heat+hot substn occ unspecif place
U47..00	[X]Exposure to smoke, fire and flames, undetermined intent
U470.00	[X]Exposure to smoke fire+flame undeterm intent occ at home
Z1B1400	Attention to dressing of burnt skin
Z1B2100	Dressing of burnt skin
Z1B2111	Burn dressing
Z1B2200	Covering burnt skin with plastic bag
ZQ3A.00	Assessment of burn injuries
ZQ3A.11	Assessment of levels of burns
