# Supplementary material for: Differing patterns in thermal injury incidence and hospitalisations among 0–4 year old children from England
Source: Burns. 2016 Nov;42(7):1609–16. doi: 10.1016/j.burns.2016.05.007 (PMC5062947; doi:10.1016/j.burns.2016.05.007)

Supplementary file 2: Description of algorithm used to identify incident thermal injury events

Full details about how incident injury events were identified is described in:

1. Baker R, Tata LJ, Kendrick D, Orton E. Identification of incident poisoning, fracture and burn events using linked primary care, secondary care and mortality data from England: implications for research and surveillance. Inj Prev. 2015. 10.1136/injuryprev-2015-041561

Table 1: Description of time-windows used to identify incident thermal injury events events(1)

**Description of time-window**	**Time-window used to identify incident thermal injury events**<br><br>**(weeks)**
**Time-window 1: From first to subsequent code in CPRD.** Time from the start date of injury event, i.e. when the first code for the injury event was recorded in primary care (CPRD). Codes recorded in primary care within this time-window were considered the same injury event.	3
**Time-window 2: From first code in HES to subsequent code in CPRD.** Time from the hospital discharge date. Used in cases where the first code of the injury event was a hospital admission recorded in HES. Codes recorded in primary care within this time-window were considered the same injury event.	8
**Time-window 3: From first CPRD or HES record to subsequent code in HES.**<br>• Time from the start date of injury event if first code recorded in CPRD<br>OR<br>• Time from the hospital discharge date if first code recorded in HES.<br><br>After this time-window, a hospital admission would be considered a new injury event.	6
**Time-window 4 (burns only): From first CPRD or HES record to procedural codes for skin grafts.**<br>Time from the start date of injury event (whether recorded in CPRD or HES) to codes for skin grafts recorded in CPRD or HES.	104

<u>Figure 1:</u> Algorithm used to define incident thermal injury events when using linked CPRD-HES data

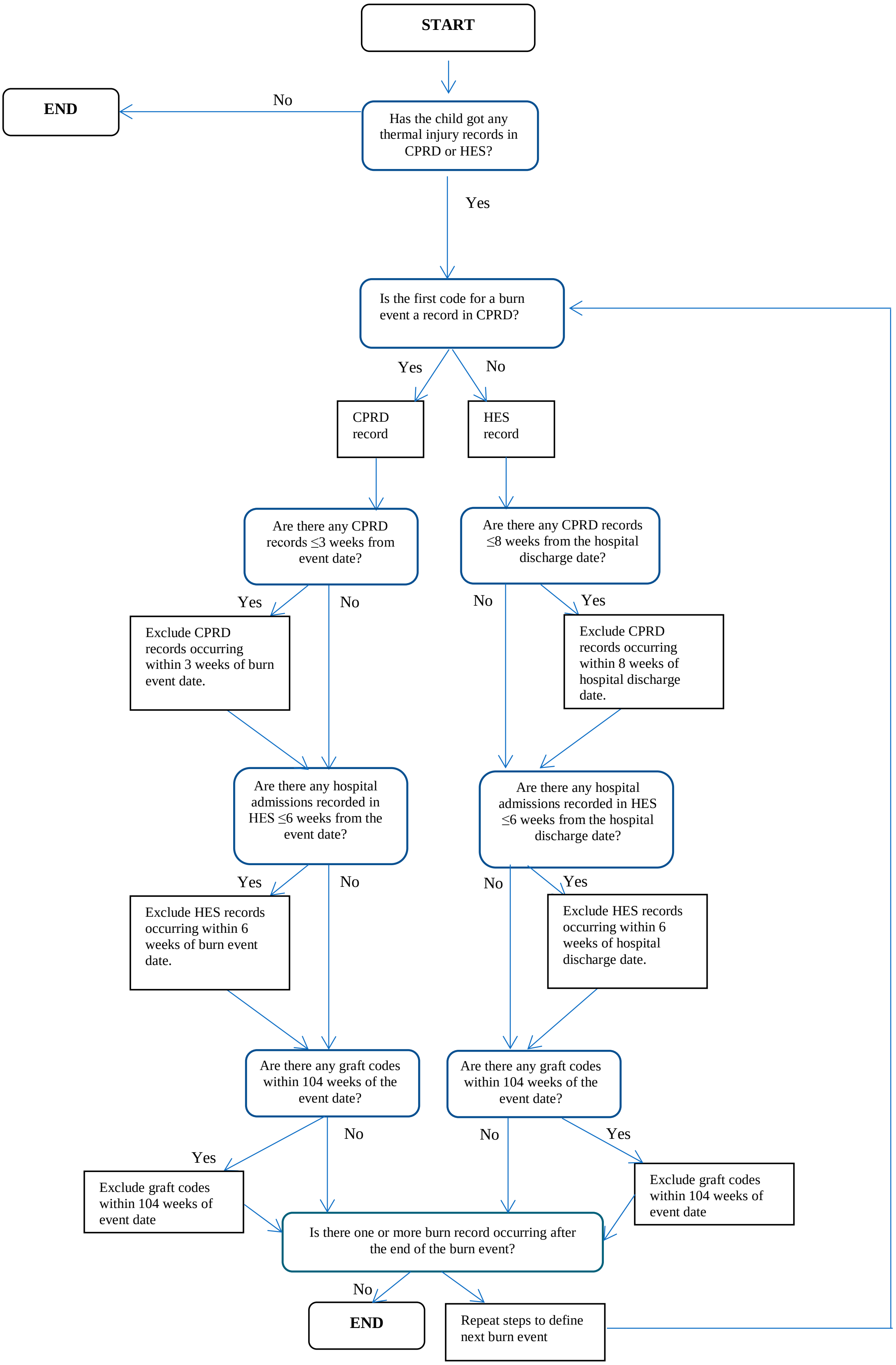

Supplement: Supplementary file 2 [file mmc2.pdf]
