# Supplementary material for: Differing patterns in thermal injury incidence and hospitalisations among 0–4 year old children from England
Source: Burns. 2016 Nov;42(7):1609–16. doi: 10.1016/j.burns.2016.05.007 (PMC5062947; doi:10.1016/j.burns.2016.05.007)

Supplementary file 3: Thermal injury incidence, hospitalisations, and hospitalisations requiring admission for 72 hours or more, according to child age

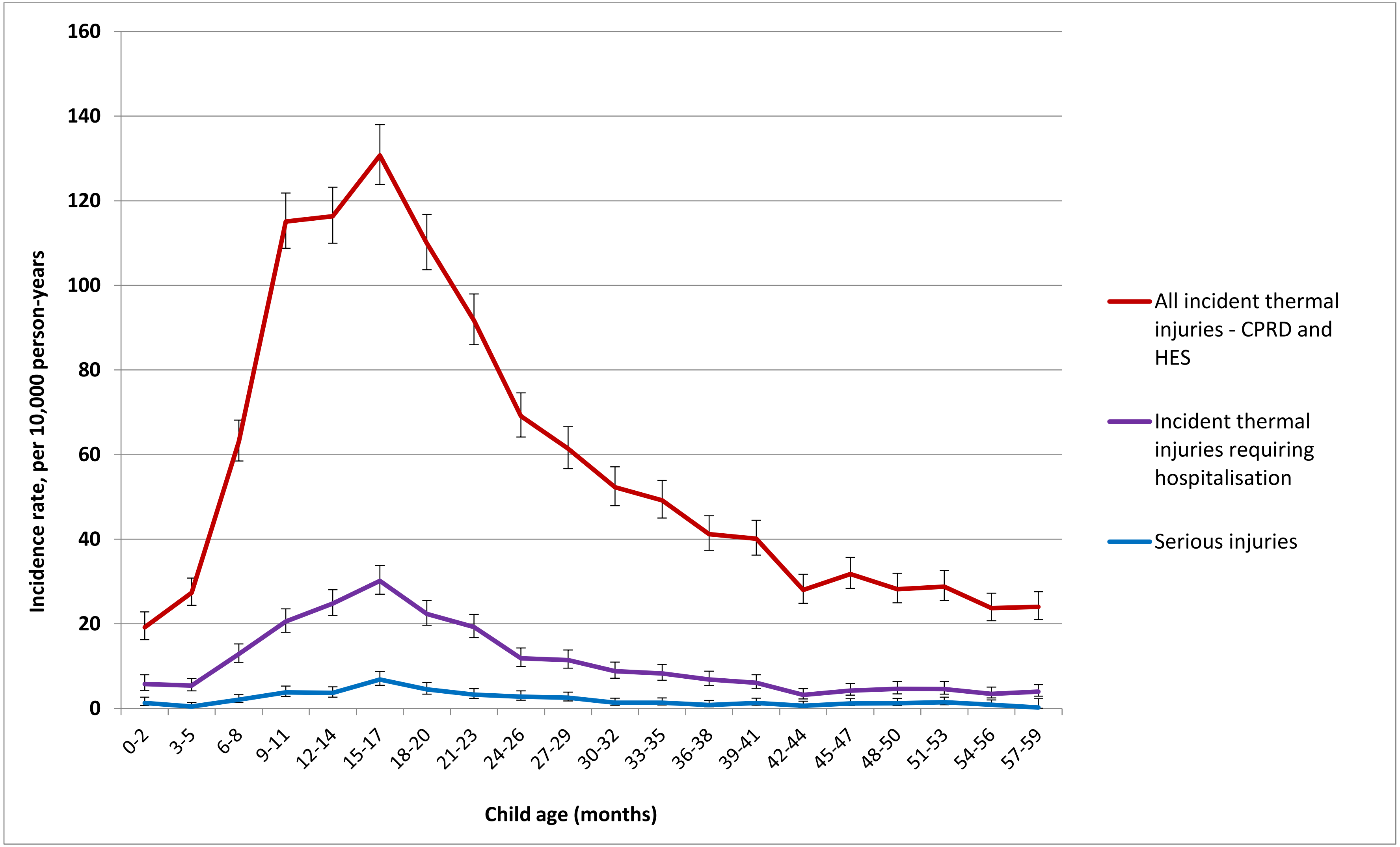

Supplement: Supplementary file 3 [file mmc3.pdf]
